# Intrathecal immune reactivity against Measles-, Rubella-, and Varicella Zoster viruses is associated with cerebrospinal fluid inflammation in multiple sclerosis

**DOI:** 10.1177/13524585241279645

**Published:** 2024-10-08

**Authors:** Benjamin Vlad, Stephan Neidhart, Marc Hilty, Klara Asplund Högelin, Ina Reichen, Mario Ziegler, Mohsen Khademi, Andreas Lutterotti, Axel Regeniter, Roland Martin, Faiez Al Nimer, Ilijas Jelcic

**Affiliations:** Neuroimmunology and Multiple Sclerosis Research Section, Department of Neurology, University of Zurich and University Hospital, Zurich, Switzerland; Neuroimmunology and Multiple Sclerosis Research Section, Department of Neurology, University of Zurich and University Hospital, Zurich, Switzerland; Swiss Epilepsy Center (Klinik Lengg), Zurich, Switzerland; Neuroimmunology and Multiple Sclerosis Research Section, Department of Neurology, University of Zurich and University Hospital, Zurich, Switzerland; Department of Neurology, Hirslanden Klinik Zurich, Zurich, Switzerland; Center for Molecular Medicine, Neuroimmunology Unit, Department of Clinical Neuroscience, Karolinska Institutet, Stockholm, Sweden; Neuroimmunology and Multiple Sclerosis Research Section, Department of Neurology, University of Zurich and University Hospital, Zurich, Switzerland; Neuroimmunology Outpatient Clinic, Center for Multiple Sclerosis, Neurocenter Bellevue, Zurich, Switzerland; Neuroimmunology and Multiple Sclerosis Research Section, Department of Neurology, University of Zurich and University Hospital, Zurich, Switzerland; Center for Molecular Medicine, Neuroimmunology Unit, Department of Clinical Neuroscience, Karolinska Institutet, Stockholm, Sweden; Neuroimmunology and Multiple Sclerosis Research Section, Department of Neurology, University of Zurich and University Hospital, Zurich, Switzerland; Clinical Research Priority Program MS (CRPP), PrecisionMS of the University of Zurich, Zurich, Switzerland; Neuroimmunology Outpatient Clinic, Center for Multiple Sclerosis, Neurocenter Bellevue, Zurich, Switzerland; Infectious Disease Serology and Immunology, Medica Medizinische Laboratorien Dr. F. Kaeppeli AG, Zurich, Switzerland; Neuroimmunology and Multiple Sclerosis Research Section, Department of Neurology, University of Zurich and University Hospital, Zurich, Switzerland; Clinical Research Priority Program MS (CRPP), PrecisionMS of the University of Zurich, Zurich, Switzerland; Center for Molecular Medicine, Therapeutic Immune Design Unit, Department of Clinical Neuroscience, Karolinska Institutet, Stockholm, Sweden; Center for Molecular Medicine, Neuroimmunology Unit, Department of Clinical Neuroscience, Karolinska Institutet, Stockholm, Sweden; Neuroimmunology and Multiple Sclerosis Research Section, Department of Neurology, University of Zurich and University Hospital, Zurich, Switzerland; Clinical Research Priority Program MS (CRPP), PrecisionMS of the University of Zurich, Zurich, Switzerland; Neuroimmunology Outpatient Clinic, Center for Multiple Sclerosis, Neurocenter Bellevue, Zurich, Switzerland

**Keywords:** Antibodies, viral/cerebrospinal fluid*, biomarkers/cerebrospinal fluid, herpesvirus 3, human/immunology*, humans, measles virus/immunology*, multiple sclerosis/cerebrospinal fluid*, multiple sclerosis/virology, Rubella virus/immunology*

## Abstract

**Background/Objectives:**

We aimed to determine in multiple sclerosis (MS) whether intrathecal immunoglobulin G (IgG) production against measles- (M), rubella- (R), and varicella zoster (Z) viruses, which is called MRZ reaction (MRZR) and considered the most specific soluble biomarker for MS, is associated with demographic and basic cerebrospinal fluid (CSF) parameters reflecting inflammation.

**Methods:**

We analyzed the presence of positive MRZR and associations with demographic and clinical routine CSF parameters in 513 patients with MS and 182 non-MS patients.

**Results:**

Comparing MS patients versus non-MS patients, positive MRZR (38.8% versus 2.2%; specificity 97.8%; positive likelihood ratio, PLR 17.7) had a better specificity and PLR for MS than CSF-specific OCB (89.5% versus 22.0%; specificity 78.0%; PLR 4.1). A positive MRZR in MS patients was associated with female sex (*p* = 0.0001), pleocytosis (*p* < 0.0001), higher frequency of presence of plasma cells in CSF (*p* = 0.0248), normal CSF/serum albumin ratio (*p* = 0.0005), and intrathecal production of total IgG or CSF-specific OCB (both *p* < 0.0001), but not with intrathecal production of total IgA or IgM.

**Conclusions:**

This study confirms the MRZR as a highly specific marker of MS and shows that MRZR-positive MS patients more frequently are female and show inflammatory changes of basic CSF parameters than MRZR-negative MS patients.

## Introduction

Multiple sclerosis (MS) is the most frequent chronic inflammatory disorder of the central nervous system (CNS) and one of the most common causes of neurological disability in young adults.^1^ Several genetic and environmental risk factors have been identified,^2,3^ and females are affected two to three times more often than males for until now unknown reasons.^2,4^ T cells and B cells are thought to be major players in the initiation and maintenance of CNS damage, and an autoreactive CD4 + T cell repertoire and several MS autoantigens have been described.^5,6^ However, important challenges remain in diagnosing MS correctly, predicting the course and severity, and choosing the right therapeutic option. According to the 2017 revised McDonald criteria, the diagnosis of MS is based on the principle of dissemination of typical clinical events and/or typical MRI lesions in space and time, the detection of cerebrospinal fluid (CSF)-specific oligoclonal bands (OCBs), and importantly the exclusion of differential diagnoses.^7^ OCBs are very sensitive in detecting intrathecal immunoglobulin G (IgG) synthesis as a typical part of the MS-related inflammation in CSF and were incorporated into MS diagnostic criteria as until now the only body fluid biomarker. However, they lack specificity, as various other MS-mimicking autoimmune and infectious disorders may also show CSF-specific OCB.^8–11^

The most specific CSF biomarker to date is the presence of intrathecal, polyspecific production of IgG antibodies reactive to a set of viruses, that is, measles virus (M), rubella virus (R), and varicella zoster virus (Z), and this reactivity pattern is therefore referred to as positive MRZ reaction (MRZR).^8,12^ The role of this intrathecal, polyspecific antiviral humoral immune response in the pathophysiology and dynamics of MS disease is still poorly understood, but the detection of a positive MRZR helps in differentiating MS from MS-mimics.^8–10^ The MRZR is widely used in routine clinical practice for CSF diagnostics when diagnosing MS in German-speaking countries, but its use has only slowly been adopted outside these countries despite its robust and highly reproducible specificity for MS.

With this work, we aim to analyze differences between MRZR-positive (MRZR^pos^) and MRZR-negative (MRZR^neg^) patients with regard to demographic characteristics and basic CSF parameters analyzed in clinical routine.

## Material and methods

### Patients

Lumbar puncture (LP) results from 513 MS patients of two centers, a Swiss cohort from the University Hospital Zurich (*n* = 354) and a Swedish cohort from the Karolinska Institutet Stockholm (*n* = 159), were retrospectively collected and anonymized. In addition, available results of MRZR from Swiss control patients (*n* = 182) with other CNS inflammatory disease (OCID, *n* = 93), extra-CNS inflammatory disease (ECID, *n* = 32), or non-inflammatory disease of the CNS (NID, *n* = 57), who received LP as part of the clinical routine work-up, were collected. MS patients had either relapsing-remitting (RRMS, *n* = 439; 234 in relapse, 205 in remission) or progressive (PMS, *n* = 73; 34 with secondary progressive MS (SPMS), 40 with primary progressive MS (PPMS)) disease course, all fulfilling the 2017 revised McDonald diagnostic criteria of MS.^7^ Notably, 77.4% of Swiss patients and 100% of Swedish patients were therapy-naïve (Supplementary Table 1). Informed consent was obtained from all patients or relatives. The Swedish Ethical Review Authority approved the study procedures (EC-No. 2009/2107-31/2). Since data of Swiss patients were anonymized, the local Cantonal Ethics Committee stated that the research project does not fall within the scope of the Human Research Act (HRA), and therefore, an authorization from the ethics committee was not required (BASEC Nr. Req-2022-01134).

### Cytological examination

Cytological examinations of the CSF were routinely obtained after a standardized protocol following the recommendations of the German Society of CSF Diagnostics and Clinical Neurochemistry (DGLN e.V.)^13^ as previously described.^10^ In a subgroup of patients with pleocytosis from the Swiss cohort, CSF-infiltrating cells were differentiated into respective leukocyte subpopulations.

### Evaluation of blood–CSF barrier function and humoral immune response

Concentrations of albumin, total IgG, total IgA, and total IgM in CSF and serum of Swiss and Swedish patients were quantified by immunonephelometry (Atellica NEPH 630 System, Siemens Healthineers, Switzerland) and their ratios calculated (Q_Alb_, Q_IgG_, Q_IgA_, and Q_IgM_, respectively). The function of the blood–CSF barrier (BCSFB) was assessed using Q_Alb_. The upper reference limit of Q_Alb_, Q_Lim_, was calculated age-dependent as (4 + (a/15))×10^–^3 according to Reiber,^14^ with “a” representing the patient’s age. Dysfunction of the BCSFB was defined as Q_Alb_ > QLim. The relative intrathecal fraction of IgG, IgA, and IgM, respectively (IgG_IF_, IgA_IF_, and IgM_IF_), was calculated according to Reiber.^15^ IgG_IF_, IgA_IF_, and/or IgM_IF_ > 0% indicated intrathecal synthesis of IgG, IgA, and/or IgM, respectively. OCBs were detected by isoelectric focusing (IEF) on agarose gels and immunoblotting using IgG-specific antibodies and a semi-automated approach (Swiss patients: Interlab G26, Interlab/Sebia, Rome, Italy; Swedish patients: Sebia, Evry, France). OCB patterns were evaluated according to international consensus criteria^16^ and were separated into “CSF-specific” or “not CSF-specific.”

### MRZ reaction

IgG antibodies against measles- (M), rubella- (R), and varicella zoster (Z) virus were measured in two different assays: Serum and CSF from 122 patients were analyzed with ELISA kits from Virion/Serion GmbH (Ruwag Bettlach, Switzerland) and fully automated ELISA processing (assay 1; four-plate ELISA processing system DSX, Dynex Technologies, Inc./Ruwag Bettlach, Switzerland). Serum and CSF from 391 patients were analyzed using commercial ELISA kits and fully automated ELISA processing from Euroimmun AG (assay 2; Euroimmun AG, Kriens, Switzerland). A total of 35 samples of CSF and serum were analyzed in both ELISA assays to determine inter-assay variability and reproducibility. The virus-specific CSF/serum antibody index (CAIspec) was calculated as previously described.^15^ CAI_spec_ ⩾ 1.5 indicated intrathecal synthesis of virus-specific antibodies. MRZR was interpreted as positive according to Reiber et al.,^17^ if polyclonal intrathecal production of antibodies against ⩾ 2 of the 3 antigens measles (M), rubella (R), and zoster (Z) was detectable.^12^

### Statistics

Differences in age and disease duration were compared with the Kruskal–Wallis test. Differences in frequency of qualitative parameters were compared with either Fisher’s exact or Chi-square test, depending on the sample size. Differences in mean values of quantitative parameters were tested for normal distribution with the Shapiro–Wilk test and calculated using the Mann–Whitney U test or t-test, accordingly. Sensitivity, specificity, positive likelihood ratio (PLR), and negative likelihood ratio (NLR) with 95% confidence intervals (95% CI) of positive MRZR were analyzed to estimate its value in distinguishing MS from controls.

## Results

### Demographic features and frequency of positive MRZR in MS patients and controls

To assess inter-assay variability and reproducibility of the MRZR, 35 samples of CSF and serum from Swiss MS patients were measured in both assays 1 and 2. For all three CAI (M-CAI, R-CAI, and Z-CAI, respectively), interpretation varied only in a single case (inter-assay variability of 2.9%). Overall interpretation of the MRZR was identical in all 35 patients (inter-assay variability of 0%), of which 18/35 had a positive MRZR (Supplementary Table 2).

MS patients from the Swedish cohort were slightly older at LP than those from the Swiss cohort (*p* = 0.015) but did not differ in terms of sex, disease duration or distribution of MS subtypes, and most importantly in the frequency of positive MRZR (Supplementary Table 1). Notably, 84.4% of all MS patients (77.4% of Swiss MS patients and all of the Swedish MS patients) were therapy-naive (Supplementary Table 1). Compared with therapy-naive MS patients, disease-modifying therapy (DMT)-treated MS patients showed a significantly higher age and a longer disease duration at LP, a higher percentage of PMS than RRMS patients, as well as a significantly lower CSF white cell count (WCC), more frequently a normal CSF WCC and a lower intensity and frequency of intrathecal IgG synthesis according to Reiber,^15^ but no significant differences in the frequency of CSF-specific OCBs as a more sensitive marker of intrathecal IgG production and also no significant differences in the frequencies of positive MRZR or BCSFB dysfunction (Supplementary Table 3).

RRMS and PMS patients differed in terms of age at LP (*p* < 0.001), frequency of female sex (*p* = 0.011), disease duration in months (*p* < 0.001), and history of disease-modifying therapy (*p* < 0.001), but there was no significant difference in frequency of positive MRZR (*p* = 0.958) (Table 1). Nonetheless, a positive M-CAI was less frequent in RRMS patients (*p* = 0.003), while the frequency of positive R-CAI (*p* = 0.410) and positive Z-CAI (*p* = 0.453) did not differ significantly between the groups. The same effects regarding the frequency of single virus-specific CAI positivity were observed in group comparison when analyzing frequencies of positive single virus CAI values and MRZR in SPMS and PPMS separately (Supplementary Table 4).

**Table 1.** Comparison of demographic features and frequency of positive single virus-specific CSF/serum antibody index (CAI) values and positive MRZ reaction in patients with relapsing-remitting MS (RRMS) and progressive MS (PMS). Intrathecal production of IgG against a specific viral antigen is present, if measles (M)-, rubella (R)-, or zoster (Z)-specific CAI is ⩾ 1.5. Positive MRZ reaction is defined as intrathecal production of IgG reactive against at least two of three antigens (M, R, and Z), that is, M + R or M + Z or R + Z or M + R + Z. Bold values indicate statistical significance (*p* < 0.05).

Parameter	Overall	RRMS	PMS	*p* value
N	513	439	74	
Age at LP, median [Q1, Q3]	35.0 [29.0, 44.0]	34.0 [28.0, 41.0]	49.0 [39.0, 54.0]	**<0.001**
Female sex, n/*N* (%)	334/513 (65.1%)	296/439 (67.4%)	38/74 (51.4%)	**0.011**
Disease duration in months, median [Q1, Q3]	9.0 [1.0, 49.0]	5.0 [0.0, 47.0]	106.0 [48.0, 215.0]	**<0.001**
Therapy-naïve*, n/*N* (%)	433/513 (84.4%)	391/439 (89.1%)	42/74 (56.8%)	**<0.001**
Positive M-CAI, n/*N* (%)	194/513 (37.8%)	154/439 (35.1%)	40/74 (54.1%)	**0.003**
Positive R-CAI, n/*N* (%)	206/513 (40.2%)	180/439 (41.0%)	26/74 (35.1%)	0.410
Positive Z-CAI, n/*N* (%)	211/513 (41.1%)	184/439 (41.9%)	27/74 (36.5%)	0.453
Positive MRZ reaction, n/*N* (%)	199/513 (38.8%)	171/439 (39.0%)	28/74 (37.8%)	0.958

Q1 and Q3—first quartile and third quartile.

* Therapy-naïve is defined as untreated patients or patients who have previously been treated with any of the further listed medications and have been off therapy for the respective time periods: (i) corticosteroids off for at least 4 weeks before LP, (ii) glatiramer acetate, interferon-beta, and dimethylfumarate off for at least 2 months before LP, (iii) teriflunomide, fingolimod, and natalizumab off for at least 3 months before LP, (iv) ocrelizumab, rituximab, and mitoxantrone off for at least 12 months before LP, and (v) aHSCT off for at least 2 years before LP.

Notably, 38.8% of all MS patients, but only 2.2% of non-MS controls had positive MRZR (Table 2). Among non-MS controls, 2.2% of OCID patients, 6.3% of ECID patients, and none of NID patients showed positive MRZR (**Supplementary Table 5**). Positive MRZR displayed an overall sensitivity of 38.8%, a specificity of 97.8%, a PLR of 17.65, and an NLR of 0.63 for the diagnosis of MS (Table 2, **Supplementary Table 6**). In comparison, CSF-specific OCBs were present in 89.5% of all MS patients but could also be detected in 22.0% of non-MS patients (Table 2, **Supplementary Table 7**). Accordingly, CSF-specific OCB displayed a stronger sensitivity of 89.5% and NLR of 0.13, but a much weaker PLR of 4.07 and weaker specificity of 78.0% than positive MRZR. Comparable effects were obtained in the respective analysis of each OCID, ECID, and NID patient (**Supplementary Tables 6 and 7**). Comparing MS patients versus non-MS patients, double-positivity of MRZR and CSF-specific OCB (195/513 (38.0%) versus 4/182 (2.2%) specificity 97.8%; PLR 17.3) also showed a significantly better specificity for MS (*p* = 0.0001) and better PLR for MS than CSF-specific OCB alone (459/513 (89.5%) versus 40/182 (22.0%) specificity 78.0%; PLR 4.1). A PLR of 17–18 increases the likelihood of disease after the test by 54%–55%, whereas a PLR of 4 makes much a smaller increase (+25%) in the likelihood.^18^

**Table 2.** Frequency of positive MRZ reaction and CSF-specific OCB among MS patients and non-MS patients and corresponding sensitivity, specificity, positive likelihood ratio (PLR), and negative likelihood ratio (NLR) of positive MRZ reaction and CSF-specific OCB in MS.

Parameter	MS patients, n/*N* (%)	Non-MS patients, n/*N* (%)	Sensitivity (95% CI)	Specificity (95% CI)	PLR (95% CI)	NLR (95% CI)
Positive MRZ reaction	199/513 (38.8%)	4/182 (2.2%)	38.8% (34.6–43.2)	97.8% (94.5–99.4)	17.65 (6.66–46.80)	0.63 (0.58–0.67)
CSF-specific OCB	459/513 (89.5%)	40/182 (22.0%)	89.5% (86.5–92.0)	78.0% (71.3–83.8)	4.07 (3.09–5.36)	0.13 (0.10–0.18)

### Differences in demographic data between MS patients with positive and negative MRZR

**Figure 1.**
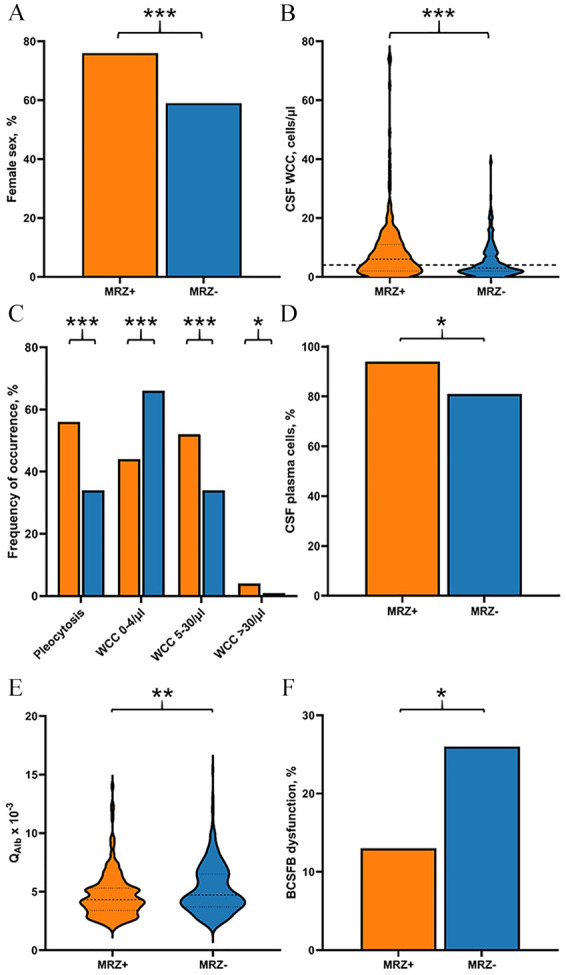
Differences in sex proportion and basic CSF parameters between MS patients with positive and negative MRZ reaction. Depicted are the differences between MS patients with positive MRZ reaction (MRZ+) and MS patients with negative MRZ reaction (MRZ-) regarding (A) sex proportion, (B) CSF white cell count (WCC) in cells/µL, (C) frequency of pleocytosis, WCC 0–4/µL, WCC 5–30/µL, WCC > 30/µL (with dashed line delimiting cut-off for normal CSF cell numbers, i.e., 4/µL), (D) frequency of samples with detection of plasma cells in CSF in %, (E) CSF/serum albumin quotient (Q_Alb_ x 10^-3^), and (F) frequency of samples with blood–CSF barrier (BCFSB) dysfunction. *p < 0.05, **p < 0.01, ***p < 0.001.

MRZR^pos^ MS patients were slightly older at the time of LP than MRZR^neg^ MS patients (*p* = 0.014), but there was no significant difference in terms of disease duration in months (*p* = 0.493) or history of DMT (*p* = 0.894) (Table 3). Female sex was strongly associated with positive MRZR (OR 2.16, 95% CI 1.46–3.20, *p* < 0.001) (**Figure 1A**). When comparing MRZR^pos^ and MRZR^neg^ MS patients (i) without previous treatment (Supplementary Table 8), (ii) with relapsing-remitting disease course only (**Supplementary Table 9**), or (iii) with progressive disease course only, female sex was again significantly more frequent in patients with positive MRZR in each subgroup. MRZR^pos^ RRMS patients were slightly older at the time of LP (*p* = 0.007) and had a marginally longer disease duration in months (*p* = 0.035) compared with MRZ^neg^ RRMS patients, but this was not the case for untreated MS patients or PMS patients (**Supplementary Tables 8 and 9**).

**Table 3.** Comparison of demographic features and basic CSF parameters between MS patients with positive and negative MRZ reaction. Bold values indicate statistical significance (*p* < 0.05).

	Overall	Positive MRZ reaction	Negative MRZ reaction	*p* Value
*n*/*N* (%)	513/513 (100%)	199/513 (38.8%)	314/513 (61.2%)	**<0.001**
Age at LP, median [Q1, Q3]	35.0 [29.0, 44.0]	37.0 [29.5, 46.5]	34.0 [28.0, 43.0]	**0.014**
Female sex, *n*/*N* (%)	334/513 (65.1%)	150/199 (75.4%)	184/314 (58.6%)	**<0.001**
Disease duration in months, median [Q1, Q3]	3.0 [0.0, 23.5]	4.0 [1.0, 37.0]	2.0 [0.0, 22.0]	0.493
History of DMT, *n*/N (%)	80/513 (15.6%)	30/199 (15.1%)	50/314 (15.9%)	0.894
CSF WCC, mean (±SD)	6.5 (±8.4)	8.7 (±10.9)	5.1 (±5.8)	**<0.001**
Pleocytosis, *n*/*N* (%)	219/511 (42.9%)	112/199 (56.3%)	107/312 (34.3%)	**<0.001**
- WCC 0–4/µL, *n*/*N* (%)	292/513 (57.1%)	87/199 (43.7%)	205/312 (65.7%)	**<0.001**
- WCC 5–30/µL, *n*/*N* (%)	209/511 (40.9%)	104/199 (52.3%)	105/312 (33.7%)	**<0.001**
- WCC > 30/µL, *n*/*N* (%)	10/513 (2.0%)	8/199 (4.0%)	2/312 (0.6%)	**0.016**
BCSFB dysfunction, *n*/*N* (%)	105/513 (20.5%)	25/199 (12.6%)	80/314 (25.5%)	**0.001**
Q_Alb_, mean (±SD)	5.0 (±2.0)	4.7 (±1.9)	5.2 (±2.1)	**0.004**
Q_IgG_, mean (±SD)	4.4 (±2.7)	5.5 (±3.1)	3.7 (±2.1)	**<0.001**
Q_IgA_, mean (±SD)	1.7 (±1.4)	1.6 (±1.1)	1.7 (±1.6)	0.315
Q_IgM_, mean (±SD)	0.7 (±1.2)	0.7 (±1.4)	0.7 (±1.1)	0.973
Intrathecal synthesis of total IgG (Reiber), *n*/*N* (%)	289/513 (56.3%)	161/199 (80.9%)	128/314 (40.8%)	**<0.001**
Intrathecal synthesis of total IgA (Reiber), *n*/*N* (%)	32/350 (9.1%)	15/130 (11.5%)	17/220 (7.7%)	0.316
Intrathecal synthesis of total IgM (Reiber), *n*/*N* (%)	70/350 (20.0%)	31/130 (23.8%)	39/220 (17.7%)	0.213
CSF-specific OCB, *n*/*N* (%)	445/513 (86.7%)	195/199 (98.0%)	250/314 (79.6%)	**<0.001**

Q1, Q3—first quartile and third quartile.

SD—standard deviation.

### Differences in basic CSF data between MS patients with positive and negative MRZR

In the whole MS cohort, positive MRZR was associated with pleocytosis (OR 2.47, 95% CI 1.71–3.55, *p* < 0.001) and a higher mean WCC (*p* < 0.001) (Table 3, **Figure 1(B) and (C)**). Accordingly, a WCC of 0–4/µL was most frequent in MRZR^neg^ patients (*p* < 0.001), while MRZR^pos^ patients had more frequent WCC 5–30/µL (*p* < 0.001) or WCC > 30/µL (*p* = 0.016) (**Figure 1(C)**).

CSF white cell differentiation in 135 untreated patients with WCC > 4/µL showed a higher frequency of samples with detection of plasma cells in MRZR^pos^ patients (OR 3.85, 95% CI 1.18–12.51, *p* = 0.021, **Figure 1(D)**), while there were no significant differences in frequency of CSF samples with detection of neutrophils, eosinophils, basophils, or macrophages between MRZR^pos^ and MRZR^neg^ MS patients (**Supplementary** Table 10).

BCSFB dysfunction as indicated by increased Q_Alb_ was significantly less frequent in MRZR^pos^ MS patients (OR 2.38, 95% CI 1.46–3.88, *p* = 0.001), and their mean Q_Alb_ was accordingly lower (*p* = 0.004) (Table 3, **Figure 1(E) and 1(F)**).

**Figure 2.**
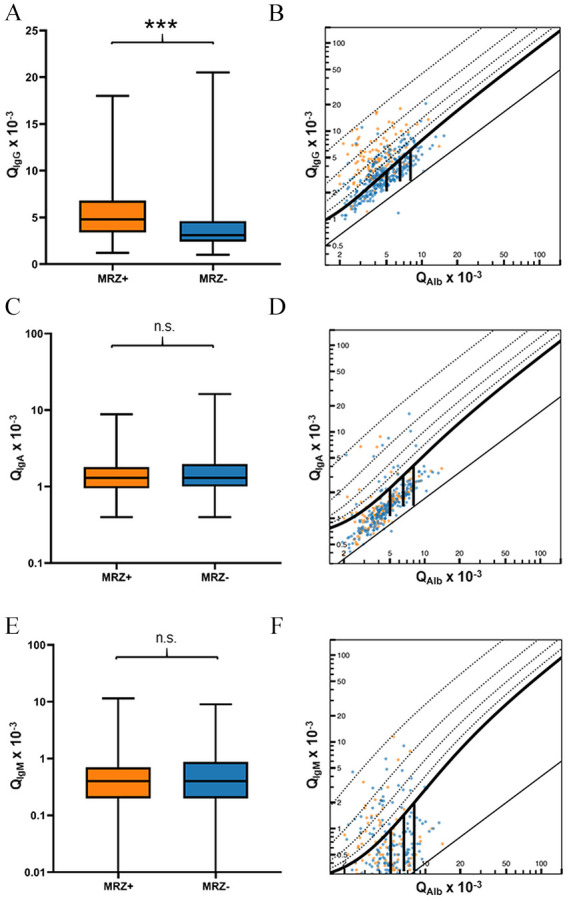
Differences in intrathecal production of IgG, IgA, or IgM between MS patients with positive and negative MRZ reaction. Depicted are the differences between MS patients with positive MRZ reaction (MRZ+) and MS patients with negative MRZ reaction (MRZ–) regarding CSF/serum ratios of (A) IgG (Q_IgG_), (C) IgA (Q_IgA_), and (E) IgM (Q_IgM_), and Reiber diagrams showing intrathecal synthesis of (B) IgG, (D) IgA, and (F) IgM. ***p < 0.001, n.s. = not significant.

**Figure 3.**
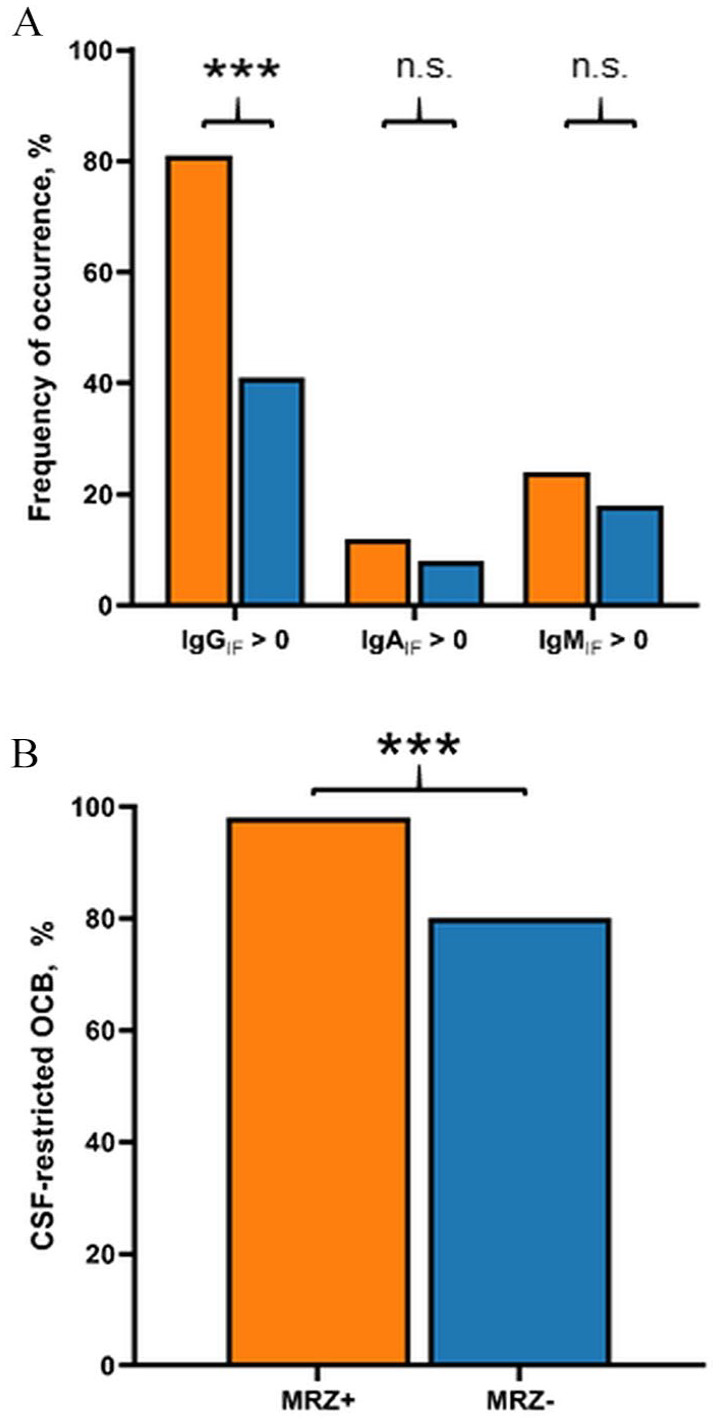
Differences in occurrence of (A) intrathecal synthesis of IgG (IgG_IF_ > 0), IgA (IgA_IF_ > 0), or IgM (IgM_IF_ > 0) and (B) CSF-specific oligoclonal bands (OCB) between MS patients with positive MRZ reaction (MRZ+) and negative MRZ reaction (MRZ–). ***p < 0.001, n.s. = not significant.

Q_IgG_ was higher (*p* < 0.001) and intrathecal synthesis of total IgG according to Reiber15 was more frequent in MRZR^pos^ MS patients (OR 6.16, 95% CI 4.05–9.36, *p* < 0.001) (Table 3, **Figures 2A, 2B, and 3(A)**). In addition, CSF-specific OCBs were strongly associated with positive MRZR (OR 12.48, 95% CI 4.47–34.87, *p* < 0.001) (Table 3, **Figure 3B**). There were no significant differences in terms of Q_IgA_, Q_IgM_, and intrathecal synthesis of IgA or IgM according to Reiber15 (**Figures 2(C), 2(F), and 3(A)**).

If intrathecal IgG synthesis according to Reiber15 was present (Ig_IF_ > 0), the mean IgG_IF_ was higher in MRZR^pos^ patients (*p* < 0.001) (**Supplementary** Table 11). In the case of intrathecal synthesis of total IgA or IgM, mean IgA_IF_ and IgM_IF_, respectively, did not vary between MRZR^pos^ and MRZR^neg^ patients (**Supplementary** Table 11). All associations were confirmed when MRZR^pos^ and MRZR^neg^ cases were compared only among MS patients without a history of DMT ( **Supplementary Table 8** ) or only among RRMS patients ( **Supplementary Table 9** ). In PMS patients, we observed the same difference regarding higher mean values for WCC and Q_IgG_ and the higher frequency of pleocytosis, intrathecal synthesis of IgG according to Reiber,15 and CSF-specific OCB, but there was no difference in frequency of BCSFB dysfunction ( **Supplementary Table 9** ).

## Discussion

Our study confirms the MRZR as a highly specific and well-reproducible diagnostic marker for MS in the largest cohort analyzed to date including patients from two centers from two countries. Furthermore, we show significant differences in MS-typical markers of cellular and antibody-related CSF inflammation and BCSFB function between MRZR^pos^ and MRZR^neg^ MS patients, independent of MS subtype.

We here demonstrate that positive MRZR had a low sensitivity of 38.8% and very high specificity of 97.8% for the diagnosis of MS in a cohort of 513 MS patients and 182 non-MS control patients. In clinical routine, PLR and NLR are considered superior to sensitivity and specificity,^19^ and a PLR > 10 and NLR < 0.1 are usually considered meaningful.20 Positive MRZR showed a PLR of 17.65, and the NLR was 0.63. The high PLR value means that the diagnosis of MS is more than 17 times more likely correct if the MRZR is positive. The mediocre NLR value means that a negative MRZR does not exclude MS mimics. In comparison, CSF-specific OCBs were present in almost a third of control patients with other CNS or extra-CNS inflammatory diseases and hence reduced the PLR drastically while meaningfully improving the NLR. This suggests that the absence of CSF-specific OCB is useful as a “rule-out” marker, but their presence only of limited use as a “rule-in” marker for the diagnosis of MS. We therefore recommend that testing MRZR should be included in the routine CSF work-up in patients with suspected MS.

Although healthy donors may be generally useful to better define the sensitivity of a laboratory marker, we did not include healthy donors as an additional control cohort because they are known to be negative for MRZR,^8,21^ and the sensitivity of the MRZR in MS was expected to be lower than CSF-specific OCB based on previous data, which we ultimately confirmed. The inclusion of healthy donors would therefore neither change specificity, which is best defined in comparison with MS mimics, nor the sensitivity of positive MRZR for MS.

The prevalence of a positive MRZR in MS patients varies considerably in reports published over the past 30 years.^8^ While older studies from the 90s reported higher rates (67%–72%) of positive MRZR^12,17^ and a recent review^8^ from 2017 summarized past studies to a cumulative rate of positive MRZR in adult MS patients of 67.4%, more recent studies—similar to our work—show a tendency toward lower rates of positive MRZR (32%–46%).^22–24^ Briefly, possible reasons could include (i) different distribution of MS disease courses and/or subtypes among studies, (ii) different demographic features, (iii) the use of different MS diagnostic criteria or other selection biases, (iv) different ELISA methods, and (v) the introduction of mass vaccination campaigns in the 1970s and 1980s and the subsequent marked decline in the prevalence of measles- and rubella-specific serum antibodies.25 With this work, we show that the MRZR is well reproducible with two different ELISA methods and thus a robust biomarker for the diagnosis of MS—at least when using these assays. Therefore, we think that differences in current ELISA kits available for the detection of MRZR are not a major factor biasing the rates of positive MRZR among recent studies. Recently, we discovered strong associations between HLA-DR15 alleles and positive MRZR and hypothesized that differential geographical distribution of HLA-DR15 alleles, which can vary considerably (15.9%–61.0%) among MS patients, may have an additional influence on rates of positive MRZR.^26^

In the present and a more recent work, we found an association of a positive MRZR with female sex in MS (independent of HLA).^26^ This sex-specific association could be related to the in general higher rate of intrathecal IgG production in female than in male MS patients.^27^ Furthermore, humoral responses in autoimmune diseases, infectious diseases, and vaccine immunity are known to be stronger in females than in males.^28^ Reasons for these sex-dependent differences in immune functions could be the influence of sex hormones, X chromosome-encoded immune factors, sex-related epigenetic dysregulations, and/or sex-specific interactions with environmental factors.

We observed a slight difference in the prevalence of positive MRZR between the Swiss (38.8%) and the Swedish cohort (42.1%), although not statistically significant. Notably, the proportion of females in the Swedish cohort was greater than that in the Swiss cohort (71.1% vs. 62.4%). Sex distribution probably played an important role in both the present work and previous studies. The significant difference in the frequency of cases with intrathecal measles-specific IgG synthesis between Swiss and Swedish MS patients could be related to geographical and temporal variations of natural or vaccine-induced exposure to measles virus antigens. Our observation that a positive measles virus-specific CSF/serum antibody index is no longer the most frequent index within the MRZR has also been shown in more recent studies.^24,29^ The comparison of DMT-treated and untreated MS patients suggests that DMTs may reduce the CSF WCC and the intensity of intrathecal IgG production (although these effects may be also confounded by older age, longer disease duration and/or higher percentage of PMS patients), but that they have no effect on the frequencies of positive MRZR, the presence of intrathecal IgG production or BCSFB dysfunction. The possible effects of DMTs on CSF WCC and other CSF parameters are in line with previous reports.^30,31^ However, the heterogeneity of DMTs regarding the type of DMT and associated demographics within the DMT group was too great, so the specific effects of individual DMTs on the specific CSF parameters should be analyzed in larger cohorts.

The cellular and molecular mechanisms associated with a positive MRZR remain enigmatic. The polyspecific antiviral antibody response is not thought to be directly involved in pathogenic processes, and concurrent viral CNS infection with measles, rubella, and/or VZV is highly unlikely.^8,32^ According to one hypothesis, EBV infection could trigger invasion of the CNS by antibody-producing cells which are specific for antigens recognized before EBV infection and linked to high frequencies of memory B cells in peripheral blood.^33^ The temporal delay of forming EBV-specific memory immune responses after acute EBV infection could also be the reason why EBV itself is only rarely part of this polyspecific immune response, as a review of nine studies shows, in which intrathecal antibody production against EBV was found consistently less frequently (9.7%) in patients with MS than that against measles (66.4%), rubella (56.5%), or varicella zoster virus (51%) antigens.^34^ After entry into the CNS, activated B cells and/or antibody-producing cells with reactivity against measles, rubella, and/or varicella zoster viruses then may transform into long-lived plasma cells. Further studies need to clarify the reasons for the robust finding of low intrathecal EBV-specific antibody production in comparison with other viruses as found in the MRZR despite the growing evidence of a unique role of EBV in the pathoetiology of MS.^2,33,34^ Since EBV-specific antibodies in serum are found in 100% of MS patients, EBV infection is considered a “conditio sine non qua” for the development of MS.^35^ However, in comparison with the high specificity (97.8%) and low sensitivity (38.8%) of positive MRZR for the diagnosis of MS, EBV seropositivity shows high sensitivity (100.0%), but low specificity (4.8%) for MS, because 95.2% of the general population is also EBV-seropositive.^35^

One of the major findings of our work is the strong association of a positive MRZR with intrathecal IgG synthesis. Not only did patients with a positive MRZR show a significant correlation to the frequency of intrathecal IgG synthesis, but also to the intensity of intrathecal IgG production. However, the presence of a positive MRZR appears to be independent of intrathecal IgA and/or IgM synthesis. Prognostically, intrathecal IgG synthesis is considered a risk factor for increased disease activity^36^ and is associated with disability worsening.^37^ The extent to which this also applies to the presence of a positive MRZR remains to be determined.

Another important finding of our work is the stronger cellular immune response of MRZR^pos^ patients, independent of disease subtype. Varying data exist on the significance of pleocytosis at the time of diagnosis: while previous work has considered it to be a risk factor for recurrent disease activity and an unfavorable factor for disease progression,^38,39^ a recent paper has not observed a less favorable prognosis of pleocytosis at diagnosis.^40^ Notably, in the latter study, only about 18% of the 247 MS patients showed a pleocytosis, whereas, usually, a frequency of about 60% is found in the literature.^16^ Plasma cells in the CSF of MS patients were reported already in 1986. They were mentioned to be independent of disease activity and not useful as prognostic markers, but associated with pleocytosis and also intrathecal IgG synthesis.^36,41^ Our findings of higher frequency of pleocytosis, plasma cells, and intrathecal IgG synthesis in MRZR^pos^ than MRZR^neg^ patients fit to these previous reports. However, as a limitation of our study, CSF white cell differentiation was performed only in a subset of patients with pleocytosis, and the significance level of the association of positive MRZR with frequency of donors showing plasma cells in the CSF was rather low, so corrections for multiple testing would neutralize this finding. Further studies are needed to better define which CSF cell subsets are associated with a positive MRZR.

BCSFB and blood–brain barrier function are markers for CSF and/or interstitial fluid flow and dysfunction accordingly describes a reduced flow.^15^ In clinical practice, Q_Alb_ is a simple and effective method to assess barrier functionality in an age-normalized manner. Abnormally high albumin levels in CSF are associated with intrathecal markers for CNS injury, increased numbers of circulating proinflammatory T helper cells, and a distinct CSF lipidomic profile.^42^ Increased CSF albumin has also been associated with the development of brain atrophy, greater risk for disease-associated disability, and presence of spinal MS lesions.^43–45^ Our work shows a higher probability of BCSFB dysfunction and correspondingly higher albumin levels in the CSF in patients with a negative MRZR. Interestingly, CSF of patients with histopathological pattern I lesions (characterized by T cell and macrophage infiltration) was mainly characterized by evidence of intrathecal IgG synthesis and a positive MRZR, whereas both findings were scarce in CSF of patients with pattern II (additional antibody and complement deposition) or pattern III (distal oligodendrogliopathy) lesions.^46^ In addition, CSF samples of patients with pattern II and III lesions more often showed BCSFB dysfunction as defined by Q_Alb_ elevation compared with patients with pattern I lesions.^46^ Future studies should compare the localization and cellular/histopathological architecture of demyelinating lesions and other biomarkers of neurodegeneration and/or -inflammation between MRZR^pos^ and MRZR^neg^ MS patients.

While the detection of CSF-specific OCB has been a hallmark in the diagnosis of MS due to its excellent sensitivity, the MRZR is by far the most specific soluble biomarker for MS to date and can be considered robust and reproducible. The present work provides evidence for significant differences in the cellular and humoral immune response as well as BCSFB function between MRZR^pos^ and MRZR^neg^ MS patients, which could indicate different underlying pathomechanisms. Future studies are needed to analyze the impact of MRZR status on the disease course and prognosis of MS patients.

## Supplemental Material

sj-docx-1-msj-10.1177_13524585241279645 – Supplemental material for Intrathecal immune reactivity against Measles-, Rubella-, and Varicella Zoster viruses is associated with cerebrospinal fluid inflammation in multiple sclerosis Supplemental material, sj-docx-1-msj-10.1177_13524585241279645 for Intrathecal immune reactivity against Measles-, Rubella-, and Varicella Zoster viruses is associated with cerebrospinal fluid inflammation in multiple sclerosis by Benjamin Vlad, Stephan Neidhart, Marc Hilty, Klara Asplund Högelin, Ina Reichen, Mario Ziegler, Mohsen Khademi, Andreas Lutterotti, Axel Regeniter, Roland Martin, Faiez Al Nimer and Ilijas Jelcic in Multiple Sclerosis Journal
