# Supplementary material for: Intrathecal immune reactivity against Measles-, Rubella-, and Varicella Zoster viruses is associated with cerebrospinal fluid inflammation in multiple sclerosis
Source: Mult Scler. 2024 Oct 8;30(13):1598–608. doi: 10.1177/13524585241279645 (PMC11568678; doi:10.1177/13524585241279645)
Supplement: sj-docx-1-msj-10.1177_13524585241279645 – Supplemental material for Intrathecal immune reactivity against Measles-, Rubella-, and Varicella Zoster viruses is associated with cerebrospinal fluid inflammation in multiple sclerosis [file sj-docx-1-msj-10.1177_13524585241279645.docx]

**Suppl. Table 1**. Comparison of demographic features and frequency of positive single virus-specific CSF/serum antibody index (CAI) values and positive MRZ reaction between the Swiss and Swedish cohort of MS patients. Patients with relapsing-remitting MS (RRMS) and progressive MS (PMS, i.e. secondary progressive MS and primary progressive MS) were included. Intrathecal production of IgG against a specific viral antigen is present, if measles (M)-, rubella (R)- or zoster (Z)-specific CAI is ≥1.5. Positive MRZ reaction is defined as intrathecal production of IgG reactive against at least two of three antigens (M, R and Z), i.e, M+R or M+Z or R+Z or M+R+Z. Bold values indicate statistical significance (p <0.05).

| **Parameter** | **Overall** | **Swiss cohort** | **Swedish cohort** | **p value** |
| --- | --- | --- | --- | --- |
| **N** | 513 | 354 | 159 | – |
| **Age at LP, median [Q1,Q3]** | 35.0 [29.0, 44.0] | 34.0 [28.0, 43.0] | 36.0 [29.5, 47.0] | **0.015** |
| **Female sex, n/N (%)** | 334/513 (65.1%) | 221/354 (62.4%) | 113/159 (71.1%) | 0.072 |
| **Disease duration in months, median [Q1,Q3]** | 3.0  [0.0, 23.5] | 3.0 [0.0, 58.5] | 2.0 [1.0, 11.0] | 0.007 |
| **Therapy-naïve, n/N (%)*** | 433/513 (84.4%) | 274/354 (77.4%) | 159/159 (100.0%) | **<0.001** |
| **RRMS patients, n/N (%)** | 439/513  (85.6%) | 301/354  (85.0%) | 138/159  (86.8%) | 0.599 |
| **PMS patients, n/N (%)** | 74/513 (14.4%) | 53/354  (15.0%) | 21/159 (13.2%) | 0.599 |
| **Positive M-CAI, n/N (%)** | 194/513 (37.8%) | 122/354 (34.5%) | 72/159 (45.3%) | **0.025** |
| **Positive R-CAI, n/N (%)** | 206/513 (40.2%) | 146/354 (41.2%) | 60/159 (37.7%) | 0.514 |
| **Positive Z-CAI, n/N (%)** | 211/513 (41.1%) | 147/354 (41.5%) | 64/159 (40.3%) | 0.862 |
| **Positive MRZ reaction, n/N (%)** | 199/513  (38.8%) | 132/354 (37.3%) | 67/159 (42.1%) | 0.345 |

Q1, Q3 – first quartile and third quartile

* Therapy-naïve is defined as untreated patients or patients who have previously been treated with any of the further listed medications and have been off therapy for the respective time-periods: (i) corticosteroids off for at least 4 weeks before LP, (ii) glatiramer acetate, interferon-beta and dimethylfumarate off for at least 2 months before LP, (iii) teriflunomide, fingolimod and natalizumab off for at least 3 months before LP, (iv) ocrelizumab, rituximab, mitoxantrone off for at least 12 months before LP, (v) aHSCT off for at least 2 years before LP.

**Suppl. Table 2**. Inter-assay variability between assay 1 and 2 to determine frequency of positive measles (M)-, rubella (R)- and zoster (Z)-specific CSF/serum antibody index (CAI) values and positive MRZ reaction. Intrathecal production of IgG against a specific viral antigen is present, if CAI is ≥1.5. Positive MRZ reaction is defined as intrathecal production of IgG reactive against at least two of three antigens (M, R and Z), i.e, M+R or M+Z or R+Z or M+R+Z.

| **Antigen recognized by intrathecally produced IgG** | **Assay 1** | **Assay 2** | **Co-efficient of variation (%)** |
| --- | --- | --- | --- |
| **Measles (M), n/N (%)** | 15/35  (42.9%) | 14/35  (40%) | 2.9% |
| **Rubella (R), n/N (%)** | 15/35  (42.9%) | 14/35  (40%) | 2.9% |
| **Zoster (Z), n/N (%)** | 17/35  (48.6%) | 16/35  (45.7%) | 2.9% |
| **Positive MRZ reaction, n/N (%)** | 18/35  (51.4%) | 18/35 (51.4%) | 0% |

**Suppl. Table 3.** Comparison of demographic features and basic CSF parameters between therapy-naïve MS patients and MS patients treated with disease-modifying therapy (DMT) at time of lumbar puncture (LP). Bold values indicate statistical significance (p <0.05).

|  | **Overall** | **Therapy-naïve MS patients at LP** | **MS patients with DMT at LP** | **p value** |
| --- | --- | --- | --- | --- |
| **N** | 513 | 433/513 (84.4%) | 80/513 (15.6%) | **-** |
| **Age at LP, median [Q1,Q3]** | 35.0 [29.0, 44.0] | 34.0 [28.0, 43.0] | 40.0 [33.5, 48.0] | **<0.001** |
| **Female sex, n/N (%)** | 334/513 (65.1%) | 286/433 (66.1%) | 48/80 (60.0%) | 0.309 |
| **Disease duration in months, median [Q1,Q3]** | 3.0 [0.0, 23.5] | 1.0 [0.0, 13.0] | 107.0 [64.0, 195.0] | **<0.001** |
| **RRMS, n (%)** | 439/513 (85.6%) | 391/433 (90.3%) | 48/80 (60.0%) | **<0.001** |
| **PMS, n (%)** | 74/513 (14.4%) | 42/433 (9.7%) | 32/80 (40.0%) | **<0.001** |
| **CSF WCC, mean (**±**SD)** | 6.5 (±8.4) | 7.2 (±8.8) | 2.7 (±4.1) | **<0.001** |
| **Pleocytosis, n/N (%)** | 219/511 (42.9%) | 210/431 (48.7%) | 9/80 (11.3%) | **<0.001** |
| **- WCC 0-4/**μ**l, n/N (%)** | 292/511 (57.1%) | 221/431 (51.3%) | 71/80 (88.8%) | **<0.001** |
| **- WCC 5-30/**μ**l, n/N (%)** | 209/511 (40.9%) | 200/431 (46.4%) | 9/80 (11.3%) | **<0.001** |
| **- WCC >30/**μ**l, n/N (%)** | 10/511 (2.0%) | 10/431 (2.3%) | 0/80 (0.0%) | 0.375 |
| **BCSFB dysfunction, n/N (%)** | 105/513 (20.5%) | 86/433 (19.9%) | 19/80 (23.8%) | 0.451 |
| **Q_Alb_, mean (**±**SD)** | 5.0 (±2.0) | 5.0 (±2.0) | 5.0 (±2.2) | 0.778 |
| **Q_IgG_, mean (**±**SD)** | 4.4 (±2.7) | 4.5 (±2.7) | 3.8 (±2.3) | **0.017** |
| **Q_IgA_, mean (**±**SD)** | 1.7 (±1.4) | 1.7 (±1.5) | 1.6 (±1.1) | 0.668 |
| **Q_IgM_, mean (**±**SD)** | 0.7 (±1.2) | 0.8 (±1.2) | 0.5 (±1.0) | **<0.001** |
| **Intrathecal synthesis of total IgG (Reiber), n/N (%)** | 289/513 (56.3%) | 255/433 (58.9%) | 34/80 (42.5%) | **0.007** |
| **Intrathecal synthesis of total IgA (Reiber), n/N (%)** | 32/350 (9.1%) | 25/270 (9.3%) | 7/80 (8.8%) | 1.000 |
| **Intrathecal synthesis of total IgM (Reiber), n/N (%)** | 70/350 (20.0%) | 59/270 (21.9%) | 11/80 (13.8%) | 0.151 |
| **CSF-specific OCB, n/N (%)** | 445/513 (86.7%) | 377/433 (87.1%) | 68/80 (85.0%) | 0.593 |
| **Positive M-CAI, n/N (%)** | 194/513 (37.8%) | 162/433 (37.4%) | 32/80 (40.0%) | 0.707 |
| **Positive R-CAI, n/N (%)** | 206/513 (40.2%) | 180/433 (41.6%) | 26/80 (32.5%) | 0.138 |
| **Positive Z-CAI, n/N (%)** | 211/513 (41.1%) | 182/433 (42.0%) | 29/80 (36.3%) | 0.387 |
| **Positive MRZ reaction, n/N (%)** | 199/513 (38.8%) | 169/433 (39.0%) | 30/80 (37.5%) | 0.901 |

Q1, Q3 – first quartile and third quartile

SD – standard deviation

**Suppl. Table 4**. Frequency of single virus-specific CSF/serum antibody index (CAI) values and positive MRZ reaction in patients with relapsing-remitting MS (RRMS), secondary progressive MS (SPMS) and primary progressive MS (PPMS). Intrathecal production of IgG against a specific viral antigen is present, if measles (M)-, rubella (R)- or zoster (Z)-specific CAI is ≥1.5. Positive MRZ reaction is defined as intrathecal production of IgG reactive against at least two of three antigens (M, R and Z), i.e, M+R or M+Z or R+Z or M+R+Z. Bold values indicate statistical significance (p <0.05).

|  | **Overall** | **RRMS** | **SPMS** | **PPMS** | **p value** |
| --- | --- | --- | --- | --- | --- |
| **N** | 513 | 439 | 34 | 40 | **<0.001** |
| **Positive M-CAI, n/N (%)** | 194/513 (37.8%) | 154/439 (35.1%) | 18/34 (52.9%) | 22/40 (55.0%) | **0.008** |
| **Positive R-CAI, n/N (%)** | 206/513 (40.2%) | 180/439 (41.0%) | 9/34 (26.5%) | 17/40 (42.5%) | 0.238 |
| **Positive Z-CAI, n/N (%)** | 211/513 (41.1%) | 184/439 (41.9%) | 9/34 (26.5%) | 18/40 (45.0%) | 0.185 |
| **Positive MRZ reaction, n/N (%)** | 199/513 (38.8%) | 171/439 (39.0%) | 10/34 (29.4%) | 18/40 (45.0%) | 0.384 |

**Suppl. Table 5.** Diagnoses and frequency of MRZ reaction-positive of Swiss non-MS patients with other CNS inflammatory disease (OCID), extra-CNS inflammatory disease (ECID) or non-inflammatory disease of the CNS (NID).

| **Swiss Non-MS patients** | **Positive MRZ reaction, n (%)** |
| --- | --- |
| **a) OCID** | 2/93 (2.2%) |
| - Neurosarcoidosis (n=25, 1 with positive MRZ reaction) | 1/25 (4.0%) |
| - MOG antibody-associated disease (MOGAD) (n=30) | 0/30 (0.0%) |
| - aquaporin 4 antibody-positive neuromyelitis optica spectrum disorder (NMOSD) (n=5) | 0/5 (0.0%) |
| - acute demyelinating encephalomyelitis (ADEM) (n=2, 1 with positive MRZ reaction) | 1/2 (50.0%) |
| - autoimmune encephalitis (6 seronegative autoimmune encephalitis, 1 GAD antibody-associated autoimmune encephalitis, 1 GAD antibody-associated stiff-person syndrome, 1 autoimmune GFAP astrocytopathy, 1 NMDA-R-antibody-associated autoimmune encephalitis, 1 KLHL11-antibody-associated autoimmune encephalitis,) | 0/11 (0.0%) |
| - viral (meningo-)encephalitis (4 of unknown etiology, 2 HIV encephalopathy, 1 VZV-associated CNS vasculopathy) | 0/7 (0.0%) |
| - rheumatological disorders with CNS involvement (2 primary angiitis of the central nervous system, 1 neuro-Behçet's disease, 1 granulomatosis with polyangiitis, 1 of unknown origin) | 0/5 (0.0%) |
| - inflammatory eye disorders (3 non-MS optic neuritis, 1 chronic relapsing inflammatory optic neuropathy [CRION]) | 0/4 (0.0%) |
| - inflammatory headache disorder (1 transient headache and neurologic deficits with cerebrospinal fluid lymphocytosis [HaNDL syndrome]) | 0/1 (0.0%) |
| - other (2 transverse myelitis, 1 chronic inflammatory CNS disease of unknown origin) | 0/3 (0.0%) |
| **b) ECID** | 2/32 (6.3%) |
| - rheumatological disorders without CNS involvement (3 Sjögren’s syndrome [1 patient with Sjögren’s syndrome without CNS involvement with positive MRZ reaction], 1 cutaneous lupus erythematosus, 1 systemic sarcoidosis, 1 linear scleroderma, 1 spondylitis ankylosans, 1 Sjögren's syndrome) | 1/8 (12.5%) |
| - inflammatory eyes diseases (2 retinal vasculitis, 2 anterior uveitis, 1 intermediate uveitis, 1 posterior uveitis, 1 papillitis, 1 atypical inflammatory eye disease not further specified, 1 orbital apex syndrome, 1 optic perineuritis) | 0/10 (0.0%) |
| - inflammatory poly- or mononeuropathy (3 cranial polyneuritis, 1 Miller-Fisher syndrome, 1 Guillain-Barré syndrome, 2 neuralgic shoulder amyotrophy, 2 polyradikuloneuritis, 3 Bell's palsy [1 with positive MRZ reaction]) | 0/12 (0.0%) |
| - other (2 with post-COVID-19 condition) | 0/2 (0.0%) |
| **c) NID** | 0/57 (0.0%) |
| - neurovascular disorders (n=7) | 0/7 (0.0%) |
| - neurodegenerative disorders (n=3) | 0/3 (0.0%) |
| - chronic headache disorders (n=9) | 0/9 (0.0%) |
| - non-inflammatory eye disorders (n=14) | 0/14 (0.0%) |
| - non-inflammatory myelopathies (n=4) | 0/4 (0.0%) |
| - CNS tumors (n=3) | 0/3 (0.0%) |
| - CSF circulation disorders (n=3) | 0/3 (0.0%) |
| - psychiatric disorders (n=3) | 0/3 (0.0%) |
| - functional neurological disorders (n=4) | 0/4 (0.0%) |
| - other non-inflammatory diseases of the CNS (n=7) | 0/7 (0.0%) |

**Suppl. Table 6.** Frequency of positive MRZ reaction among MS patients and subgroups of non-MS patients and corresponding sensitivity, specificity, positive likelihood ratio (PLR) and negative likelihood ratio (NLR) of positive MRZ reaction in MS. Non-MS patients (a) had b) other CNS inflammatory disease (OCID), c) extra-CNS inflammatory disease (ECID) or d) non-inflammatory diseases (NID).

|  | **MS patients, n/N (%)** | **Non-MS patients, n/N (%)** | **Sensitivity (95% CI)** | **Specificity (95% CI)** | **PLR  (95% CI)** | **NLR  (95% CI)** |
| --- | --- | --- | --- | --- | --- | --- |
| **a) MS vs. Non-MS** | | | | | | |
| Positive MRZ reaction, n/N (%) | 199/513 (38.8%) | 4/182 (2.2%) | 38.8%  (34.6-43.2%) | 97.8%  (94.5-99.4%) | 17.65  (6.66-46.80) | 0.63  (0.58-0.67) |
| Negative MRZ reaction, n/N (%) | 314/513 (61.2%) | 178/182 (97.8%) |  |  |  |  |
| **b) MS vs. OCID** | | | | | | |
| Positive MRZ reaction, n/N (%) | 199/513 (38.8%) | 2/93 (2.2%) | 38.8%  (34.6-43.2%) | 97.8%  (92.5-99.7%) | 18.04  (4.56-71.36) | 0.63  (0.58-0.67) |
| Negative MRZ reaction, n/N (%) | 314/513 (61.2%) | 91/93 (97.8%) |  |  |  |  |
| **c) MS vs. ECID** | | | | | | |
| Positive MRZ reaction, n/N (%) | 199/513 (38.8%) | 2/32  (6.3%) | 38.8%  (34.6-43.2%) | 93.8%  (79.2-99.2%) | 6.21  (1.61-23.85) | 0.65  (0.58-0.73) |
| Negative MRZ reaction, n/N (%) | 314/513 (61.2%) | 30/32 (93.7%) |  |  |  |  |
| **d) MS vs. NID** | | | | | | |
| Positive MRZ reaction, n/N (%) | 199/513 (38.8%) | 0/57 (0.0%) | 38.8%  (34.6-43.2%) | 100.0%  (93.7-100.0%) | -  (-) | 0.61  (0.57-0.66) |
| Negative MRZ reaction, n/N (%) | 314/513 (61.2%) | 57/57 (100.0%) |  |  |  |  |

**Suppl. Table 7.** Frequency of CSF-specific OCB among MS patients and subgroups of non-MS patients and corresponding sensitivity, specificity, positive likelihood ratio (PLR) and negative likelihood ratio (NLR) of positive MRZ reaction in MS. Non-MS patients (a) had b) other CNS inflammatory disease (OCID), c) extra-CNS inflammatory disease (ECID) or d) non-inflammatory diseases (NID).

|  | **MS patients, n/N (%)** | **Non-MS patients, n/N (%)** | **Sensitivity (95% CI)** | **Specificity (95% CI)** | **PLR  (95% CI)** | **NLR  (95% CI)** |
| --- | --- | --- | --- | --- | --- | --- |
| **a) MS vs. Non-MS** | | | | | | |
| CSF-specific OCB present, n/N (%) | 459/513 (89.5%) | 40/182 (22.0%) | 89.5%  (86.5-92.0%) | 78.0%  (71.3-83.8%) | 4.07  (3.09-5.36) | 0.13  (0.10-0.18) |
| CSF-specific OCB absent, n/N (%) | 54/513 (10.5%) | 142/182 (78.0%) |  |  |  |  |
| **b) MS vs. OCID** | | | | | | |
| CSF-specific OCB present, n/N (%) | 459/513 (89.5%) | 30/93 (32.3%) | 89.5%  (86.5-92.0%) | 67.7%  (57.3-77.1%) | 2.77  (2.06-3.73) | 0.16  (0.12-0.21) |
| CSF-specific OCB absent, n/N (%) | 54/513 (10.5%) | 63/93 (67.7%) |  |  |  |  |
| **c) MS vs. ECID** | | | | | | |
| CSF-specific OCB present, n/N (%) | 459/513 (89.5%) | 10/32 (31.3%) | 89.5%  (86.5-92.0%) | 68.8%  (50.0-83.9%) | 2.86  (1.71-4.79) | 0.15  (0.11-0.22) |
| CSF-specific OCB absent, n/N (%) | 54/513 (10.5%) | 22/32 (68.7%) |  |  |  |  |
| **c) MS vs. NID** | | | | | | |
| CSF-specific OCB present, n/N (%) | 459/513 (89.5%) | 0/57 (0.0%) | 89.5%  (86.5-92.0%) | 100.0%  (93.7-100.0%) | -  (-) | 0.11  (0.08-0.14) |
| CSF-specific OCB absent, n/N (%) | 54/513 (10.5%) | 57/57 (100.0%) |  |  |  |  |

**Suppl. Table 8.** Comparison of demographic features and basic CSF parameters between therapy-naïve MS patients with positive and negative MRZ reaction. Bold values indicate statistical significance (p <0.05).

|  | **Overall** | **Therapy-naïve MS patients with positive MRZ reaction** | **Therapy-naïve MS patients with negative MRZ reaction** | **p value** |
| --- | --- | --- | --- | --- |
| **n/N (%)** | 433/433 (100%) | 169/433 (39.0%) | 264/433 (71.0%) | **<0.001** |
| **Age at LP, median [Q1,Q3]** | 34.0 [28.0, 43.0] | 36.0 [28.0, 47.0] | 33.0 [28.0, 41.0] | 0.059 |
| **Female sex, n/N (%)** | 286/433 (66.1%) | 129/169 (76.3%) | 157/264 (59.5%) | **<0.001** |
| **Disease duration in months, median [Q1,Q3]** | 1.0 [0.0, 12.0] | 2.0 [0.0, 12.5] | 1.0 [0.0, 10.75] | 0.394 |
| **CSF WCC, mean (**±**SD)** | 7.0 (±8.8) | 9.7 (±11.4) | 5.6 (±6.0) | **<0.001** |
| **Pleocytosis, n/N (%)** | 210/431 (48.7%) | 107/169 (63.3%) | 103/262 (39.3%) | **<0.001** |
| **- WCC 0-4/**μ**l, n/N (%)** | 221/431 (51.3%) | 62/169 (36.7%) | 159/262 (60.7%) | **0.001** |
| **- WCC 5-30/**μ**l, n/N (%)** | 200/431 (46.4%) | 99/169 (58.6%) | 101/262 (38.5%) | **<0.001** |
| **- WCC >30/**μ**l, n/N (%)** | 10/431 (2.3%) | 8/169 (4.7%) | 2/262 (0.8%) | **0.016** |
| **BCSFB dysfunction, n/N (%)** | 86/433 (19.9%) | 20/169 (11.8%) | 66/264 (25.0%) | **0.001** |
| **Q_Alb_, mean (**±**SD)** | 5.0 (±2.0) | 4.7 (±1.9) | 5.2 (±2.0) | **0.008** |
| **Q_IgG_, mean (**±**SD)** | 4.5 (±2.7) | 5.7 (±3.1) | 3.7 (±2.1) | **<0.001** |
| **Q_IgA_, mean (**±**SD)** | 1.7 (±1.5) | 1.5 (±1.0) | 1.8 (±1.7) | 0.192 |
| **Q_IgM_, mean (**±**SD)** | 0.8 (±1.2) | 0.8 (±1.4) | 0.8 (±1.1) | 0.968 |
| **Intrathecal synthesis of total IgG (Reiber), n/N (%)** | 255/433 (58.9%) | 144/169 (85.2%) | 111/264 (42.0%) | **<0.001** |
| **Intrathecal synthesis of total IgA (Reiber), n/N (%)** | 25/270 (9.3%) | 10/100 (10.0%) | 15/170 (8.8%) | 0.917 |
| **Intrathecal synthesis of total IgM (Reiber), n/N (%)** | 59/270 (21.9%) | 25/100 (25.0%) | 34/170 (20.0%) | 0.419 |
| **CSF-specific OCB, n/N (%)** | 377/433 (87.1%) | 166/169 (98.2%) | 211/264 (79.9%) | **<0.001** |

Q1, Q3 – first quartile and third quartile

SD – standard deviation

**Suppl Table 9.** Comparison of demographic features and basic CSF parameters between A) RRMS patients with positive and negative MRZ reaction and B) PMS patients with positive and negative MRZ reaction. Bold values indicate statistical significance (p <0.05).

|  | 1. **RRMS** | | | 1. **PMS** | | |
| --- | --- | --- | --- | --- | --- | --- |
|  | **Positive MRZ reaction** | **Negative MRZ reaction** | **p value** | **Positive MRZ reaction** | **Negative MRZ reaction** | **p value** |
| **N** | 171/439 (39.0%) | 268/439 (61.0%) | - | 28/74 (37.8%) | 46/74 (62.2%) | - |
| **Age at LP, median [Q1,Q3]** | 36.0 [28.0, 43.0] | 32.0 [28.0, 39.0] | **0.007** | 49.0 [40.5 53.5] | 49.5 [37.0, 57.0] | 0.845 |
| **Female gender, n/N (%)** | 133/171 (77.8%) | 163/268 (60.8%) | **<0.001** | 17/28 (60.7%) | 21/46 (45.7%) | 0.209 |
| **Disease duration in months, median [Q1,Q3]** | 2.0 [0.0, 17.0] | 1.0 [0.0, 11.0] | **0.035** | 70.0 [21.5, 99.5] | 32.5 [16.0, 124.0] | 0.763 |
| **History of DMT, n/N (%)** | 17/171 (9.9%) | 31/268 (11.6%) | 0.641 | 13/28 (46.4%) | 19/46 (41.3%) | 0.666 |
| **CSF WCC, mean (**±**SD)** | 9.4 (±11.5) | 5.7 (±2.3) | **<0.001** | 4.7 (±5.0) | 1.6 (±2.0) | **0.002** |
| **Pleocytosis, n/N (%)** | 103/171 (60.2%) | 105/267 (39.3%) | **<0.001** | 9/28 (32.1%) | 2/45 (4.4%) | **0.002** |
| **- WCC 0-4/**μ**l, n/N (%)** | 68/171 (39.8%) | 162/267 (60.7%) | **<0.001** | 19/28 (67.9%) | 43/45 (95.6%) | **0.002** |
| **- WCC 5-30/**μ**l, n/N (%)** | 95/171 (55.6%) | 103/267 (38.6%) | **<0.001** | 9/28 (32.1%) | 2/45 (4.4%) | **0.002** |
| **- WCC > 30/**μ**l, n/N (%)** | 8/171 (4.7%) | 2/267 (0.7%) | **0.007** | 0/28 (0.0%) | 0/45 (0.0%) | - |
| **BCSFB dysfunction, n/N (%)** | 19/171 (11.1%) | 68/268 (25.4%) | **<0.001** | 6/28 (21.4%) | 12/46 (26.1%) | 0.783 |
| **Q_Alb_, mean (**±**SD)** | 4.5 (±1.6) | 5.0 (±1.9) | **0.006** | 5.7 (±2.9) | 6.1 (±2.6) | 0.253 |
| **Q_IgG_, mean (**±**SD)** | 5.5 (±3.0) | 3.7 (±2.1) | **<0.001** | 5.7 (±3.8) | 3.8 (±1.8) | **0.034** |
| **Q_IgA_, mean (**±**SD)*** | 1.5 (±1.2) | 1.7 (±1.6) | 0.606 | 1.7 (±0.9) | 2.0 (±1.4) | 0.502 |
| **Q_IgM_, mean (**±**SD)*** | 0.7 (±1.4) | 0.7 (±1.1) | 0.912 | 0.7 (±1.4) | 0.7 (±0.9) | 0.540 |
| **Intrathecal synthesis of total IgG (Reiber), n/N (%)** | 146/171 (85.4%) | 117/268 (43.7%) | **<0.001** | 15/28 (53.6%) | 11/46 (±23.9%) | **0.013** |
| **Intrathecal synthesis of total IgA (Reiber), n/N (%)** | 13/110 (11.8%) | 14/190 (7.4%) | 0.194 | 2/21 (9.5%) | 1/30 (3.3%) | 0.561 |
| **Intrathecal synthesis of total IgM (Reiber), n/N (%)** | 27/110 (24.5%) | 33/190 (17.4%) | 0.134 | 4/22 (18.2%) | 6/30 (20.0%) | 0.870 |
| **CSF-specific OCB, n/N (%)** | 167/171 (97.7%) | 215/268 (80.2%) | **<0.001** | 28/28 (100.0%) | 35/46 (76.1%) | **0.005** |

*Data only from Swiss patients.

**Suppl. Table 10.** Comparison of CSF white cell populations between A) MS patients with positive and negative MRZ reaction, and B) only untreated MS patients with positive and negative MRZ reaction, in a subgroup of MS patients with pleocytosis from the Swiss cohort.

|  | 1. **All MS patients** | | | | 1. **Untreated MS patients** | | | |
| --- | --- | --- | --- | --- | --- | --- | --- | --- |
| Type of CSF immune cell population detected in CSF samples | **Overall** | **Positive MRZ reaction** | **Negative MRZ reaction** | **p value** | **Overall** | **Positive MRZ reaction** | **Negative MRZ reaction** | **p value** |
| **Frequency of CSF samples with detection of plasma cells, n/N (%)** | 118/135 (87.4%) | 64/68 (94.1%) | 54/67 (80.6%) | **0.0207** | 114/128 (89.1%) | 62/64 (96.9%) | 52/64 (81.3%) | **0.011** |
| **Frequency of CSF samples with detection of neutrophils, n/N (%)** | 26/135 (19.3%) | 14/68 (20.6%) | 12/67 (17.9%) | 0.860 | 25/128 (19.5%) | 13/64 (20.3%) | 12/64 (18.8%) | 0.824 |
| **Frequency of CSF samples with detection of eosinophils, n/N (%)** | 8/135 (5.9%) | 3/68 (4.4%) | 5/67 (7.5%) | 0.699 | 8/128 (6.3%) | 3/64 (4.7%) | 5/64 (7.8%) | 0.715 |
| **Frequency of CSF samples with detection of basophils, n/N (%)** | 3/135 (2.2%) | 2/68 (2.9%) | 1/67 (1.5%) | 0.568 | 2/128 (1.6%) | 1/64 (1.6%) | 1/64 (1.6%) | 1.000 |
| **Frequency of CSF samples with detection of macrophages, n/N (%)** | 18/135 (13.3%) | 8/68 (11.8%) | 10/67 (14.9%) | 0.774 | 18/128 (14.1%) | 8/64 (12.5%) | 10/64 (15.6%) | 0.799 |

**Suppl. Table 11.** Additional basic CSF parameters to Table 3.

|  | 1. **All MS** | | | 1. **RRMS** | | | 1. **PMS** | | |
| --- | --- | --- | --- | --- | --- | --- | --- | --- | --- |
|  | **Positive MRZ reaction** | **Negative MRZ reaction** | **p value** | **Positive MRZ reaction** | **Negative MRZ reaction** | **p value** | **Positive MRZ reaction** | **Negative MRZ reaction** | **p value** |
| **n/N** | 199/513  (38.8%) | 314/513  (61.2%) | <0.001 | 171/439  (39.0%) | 268/439  (61.0%) | <0.001 | 28/74  (37.8%) | 46/74  (62.2%) | 0.003 |
| **IgG_IF_ if > 0, mean (**±**SD)** | 43.4  (±21.7) | 22.6  (±17.2) | **<0.001** | 43.8  (±21.2) | 23.4  (±16.8) | **<0.001** | 39.4  (±25.8) | 14.4  (±19.5) | **0.009** |
| **IgA_IF_ if > 0, mean (**±**SD)*** | 30.7  (±29.0) | 41.1  (±26.8) | 0.298 | 34.0  (±29.7) | 37.7  (±24.7) | 0.616 | 9.65  (±11.5) | 31.8  (±35.7) | 0.285 |
| **IgM_IF_ if > 0, mean (**±**SD)*** | 45.4  (±26.2) | 49.2  (±25.0) | 0.537 | 44.3  (±25.1) | 49.9  (±25.4) | 0.369 | 53.2  (±36.4) | 20.1  (±28.8) | 0.069 |

*Data only from Swiss patients
